# Knock-down of hypoxia-induced carbonic anhydrases IX and XII radiosensitizes tumor cells by increasing intracellular acidosis

**DOI:** 10.3389/fonc.2012.00199

**Published:** 2013-01-07

**Authors:** Jérome Doyen, Scott K. Parks, Serge Marcié, Jacques Pouysségur, Johanna Chiche

**Affiliations:** ^1^Institute for Research on Cancer and Aging of Nice, CNRS UMR 7284, University of Nice Sophia-Antipolis,Nice, France; ^2^Department of Radiation Oncology, Centre Antoine-Lacassagne, Nice, France; ^3^Centre Scientifique de Monaco, Monaco

**Keywords:** carbonic anhydrases, hypoxia, intracellular pH, ionizing radiation, tumor growth

## Abstract

The relationship between acidosis within the tumor microenvironment and radioresistance of hypoxic tumor cells remains unclear. Previously we reported that hypoxia-induced carbonic anhydrases (CA) IX and CAXII constitute a robust intracellular pH (pH_i_)-regulating system that confers a survival advantage on hypoxic human colon carcinoma LS174Tr cells in acidic microenvironments. Here we investigate the role of acidosis, CAIX and CAXII knock-down in combination with ionizing radiation. Fibroblasts cells (-/+ CAIX) and LS174Tr cells (inducible knock-down for *ca9*/*ca12*) were analyzed for cell cycle phase distribution and survival after irradiation in extracellular pH_o_ manipulations and hypoxia (1% O_2_) exposure. Radiotherapy was used to target *ca9/ca12*-silenced LS174Tr tumors grown in *nude* mice. We found that diminishing the pH_i_-regulating capacity of fibroblasts through inhibition of Na^+^/H^+^ exchanger 1 sensitize cells to radiation-induced cell death. Secondly, the pH_i_-regulating function of CAIX plays a key protective role in irradiated fibroblasts in an acidic environment as accompanied by a reduced number of cells in the radiosensitive phases of the cell cycle. Thirdly, we demonstrate that irradiation of LS174Tr spheroids, silenced for either *ca9* or both *ca9*/*ca12*, showed a respective 50 and 75% increase in cell death as a result of a decrease in cell number in the radioresistant S phase and a disruption of CA-mediated pH_i_ regulation. Finally, LS174Tr tumor progression was strongly decreased when *ca9*/*ca12* silencing was combined with irradiation *in vivo*. These findings highlight the combinatory use of radiotherapy with targeting of the pH_i_-regulating CAs as an anti-cancer strategy.

## INTRODUCTION

Ionizing radiation is used therapeutically to induce cancer cell death, decrease distant metastasis rates, and to increase overall patient survival (Darby et al., 2011). However, radiotherapy does not efficiently target all cells of the tumor mass. Tumor cell re-population and activation of DNA repair mechanisms (ATM, γH2AX, and p53) are key components of tumor cell radioresistance (Huen and Chen, 2008). Cells in the G2/M and G1 phases of the cell cycle have been shown to be the most radiosensitive, while cells in the S phase are radioresistant (Hwang et al., 2000; Pawlik and Keyomarsi, 2004). The latter is attributed to DNA double strand breaks (DSBs) repair systems such as homologous recombination that occur in the S phase (Kastan and Bartek, 2004; Jackson and Bartek, 2009). In the 1950s, several groups established the connection between hypoxia and radioresistance in mammalian tumors (Deschner and Gray, 1959; Dewey, 1960) and oxygen levels remain the major cell radiosensitizer known to date. In well-oxygenated conditions, the free radicals generated by ionizing radiation insult react with O_2_ to form peroxy radicals that damage DNA much more efficiently than reduced free radicals (Brown, 2007). Consequently, the poorly oxygenated (hypoxic) cells of tumors are more radioresistant (Gray, 1953). These studies led to the general hypothesis that oxygen acts at a physicochemical level to improve radiation induced damage as a consequence of the high affinity between the oxygen molecule and the unpaired electron on the free radical produced by radiation.

In addition to low oxygen, increased acidification is also a hallmark of hypoxic tumors and it has been suggested to play an indirect role in the poor radioresponse of hypoxic tumors (Vaupel, 2004). In contrast, another report indicates that extracellular acidosis may enhance radiosensitivity in combination with lactate accumulation for certain cell lines (Grotius et al., 2009). However, lactate accumulation alone (in the absence of pH disruption) has also been suggested to reduce radiosensitivity of tumor cells (Quennet et al., 2006). Furthermore, the effect of intracellular pH (pH_i_) and extracellular pH (pH_o_) regulation on the efficacy of irradiation remains to be clarified.

Despite the fact that all mammalian cells are capable of protecting their cytosol from acidification through expression of membrane located transporters and exchangers including the Na^+^/H^+^ exchanger 1 (NHE-1; Pouysségur et al., 1985) and the monocarboxylate transporter 1 (MCT1; Halestrap and Price, 1999), hypoxic tumor cells have developed additional mechanisms to regulate their pH_i_ (Chiche et al., 2010b). In solid tumors, membrane-bound carbonic anhydrases (CA) IX and XII are controlled by oxygen levels *via *the hypoxia-inducible factor (HIF-1; Wykoff et al., 2000) and catalyze at the cell surface the reversible hydration of carbon dioxide (CO_2_) into a proton (H^+^) and bicarbonate (${\text{HCO}}_{3}^{-}$). Once generated, ${\text{HCO}}_{3}^{-}$ is proposed to be rapidly taken up into the cell through the Na^+^–${\text{HCO}}_{3}^{-}$ cotransporters (NBC; Romero et al., 2004; Parks et al., 2011) to sustain a slightly alkaline pH_i_ compatible with cell survival (Morgan et al., 2007; Swietach et al., 2009; Chiche et al., 2010a). Many reports correlate CAIX expression with poor patient survival in a variety of cancers (see review Supuran, 2008; Chiche et al., 2010a). The extracellular location of the CAIX active site together with its overexpression in hypoxic cancer cells compared to minimal expression in healthy cells, except in the gastro-intestinal tract and the stomach (Pastoreková et al., 1997) makes hypoxia-induced CAIX an accessible target for new anti-cancer therapy (Supuran, 2008; Morris et al., 2011). CAIX function has been clearly established to contribute to extracellular acidification (Svastová et al., 2004). In addition, studies in our laboratory have characterized CAIX and CAXII as robust pH_i_-regulating enzymes and have provided evidence that both CAIX and CAXII hold potential as new anti-cancer targets (Chiche et al., 2010a).

We analyzed the downstream effects of CAIX and CAXII activity on radiation-induced cell death to determine whether a combined therapy of irradiation and down-regulation of CAIX and CAXII would sensitize hypoxic cells to ionizing radiation. An alteration in pH_i_ regulation (either by inhibition of NHE-1 or expression of CAIX) revealed a decreased percentage in cells found in the radioresistant S phase and an *in vitro* radiosensitization that correlated with an increase in cell death. Gene silencing of *ca9* and *ca9*/*ca12* revealed *in vitro* and *in vivo* radiosensitization as a consequence of a reduction of cells in the S phase and a decrease in the pH_i_-regulating capacity of the cell.

## MATERIALS AND METHODS

### CELL CULTURE AND HYPOXIC EXPOSURE

Chinese hamster lung CCL39 fibroblasts (ATCC), CCL39-derived PS120 cells lacking NHE-1, and CAIX and CAXII, were cultured as described. Colon adenocarcinoma LS174Tr cells expressing the tetracycline (Tet) repressor were maintained in Dulbecco’s modified Eagle’s medium (DMEM) supplemented with 10% fetal calf serum (FCS) and blasticidin (10 μg/ml, Invitrogen). Incubation in hypoxia at 1% O_2_ was carried out at 37°C in 95% humidity and 5% CO_2_/94% N_2_ in a sealed anaerobic workstation (Ruskinn).

### CELL IRRADIATION

Irradiation of normoxic cells was performed in 25 cm^2^ ventilated flasks (Nunc), while irradiation of hypoxic cells was performed in 25 cm^2^ non-ventilated flasks to maintain 1% O_2_ during treatment after removal from the hypoxic workstation. Cells were irradiated 100 cm from the source with a bolus of 1.1 cm (under dishes). High energy photons were used (6 MV), delivered by a linear accelerator (PRIMUS^®^, Siemens) with a 40 cm × 40 cm posterior field. The dose rate of the PRIMUS was 300 monitor units/min and 2 Gy corresponded to 93 monitor units (18.6 s). Spheroids were irradiated with the same schedule but with an anterior field and a bolus placed at the top of the dishes.

### PLASMIDS

Full-length human *ca9* cDNA was obtained and inserted into pTREX-A (pcDNA4/TO/myc-His A; Invitrogen; p*ca9*) as described (Chiche et al., 2010a). The short-hairpin (sh) RNA-*ca9* (sh*ca9*) was obtained with oligonucleotides: forward 5′-AGTTAAGCCTAAATCAGAA-3′ and reverse 5′TTCTGATTTAGGCTTAACT-3′ and inserted into either pTER vector (also named sh*ca9*). Lentivirus particles for two independent sequences (#1 and #2) of pLKO.1-Puro shRNA targeting *ca12* (*ca12*^-^) and non-target shRNA (*ctl*; Sigma, TRCN0000116249, TRCN0000116251, and SHC002V) were used to constitutively silence *ca12*.

### STABLE TRANSGENIC CELLS

PS120 cells were transfected with p*ca9 *as described (Chiche et al., 2010a). Tet (10 μg/ml)-inducible LS174Tr cells silenced for *ca9 *(LS-sh*ca9/ctl*) combined with a constitutive silencing of *ca12* (LS-sh*ca9/ca12*^-^) were obtained as described (Chiche et al., 2010a).

### IMMUNOBLOTTING

Cells were lysed in 1.5 × SDS sample buffer. Proteins (40 μg) were separated on 7.5% SDS polyacrylamide gels and transferred onto polyvinylidene difluoride membranes (Millipore). Membranes were blotted with the M75 antibody to CAIX (Bayer), a polyclonal antibody to recombinant CAXII (Sigma), p21 (Santa Cruz), β1 integrin (Cell Signalling), and Hsp90 (Abcam). Immunoreactive bands were detected with a horseradish peroxidase-anti-mouse or anti-rabbit antibody (Promega) by ECL (Amersham Biosciences).

### CELL CYCLE ANALYSIS

Cells (3 × 10^5^) exposed to normoxia or hypoxia were washed in PBS and fixed in ice-cold 70% ethanol for a minimum of 30 min. Cells were centrifuged, washed in 38 mM sodium citrate (pH 7.4), and stained for 20 min at 37°C with 50 μg/ml propidium iodide (Sigma) and 5 μg/ml RNase A (Sigma) in sodium citrate solution. Cell cycle analysis was done by flow cytometry using a FACScan calibur (Becton Dickinson). The proportion of G1, G2/M peaks, and the S phase plateau were calculated with WinMD2 software.

### MEASURE OF RADIATION-INDUCED CELL DEATH

Fibroblasts (1 × 10^4^) were seeded onto 60 mm dishes. Once attached the medium was replaced by either ${\text{HCO}}_{3}^{-}$-free or 10 mM ${\text{HCO}}_{3}^{-}$-containing DMEM buffered at an extracellular pH (also named outside pH, pH_o_) of 7.0 (30 mM MES) or at pH_o_ 7.5 (30 mM HEPES), supplemented with 10% dialyzed serum, hypoxanthine 0.1 mM, and uridine triphosphate 0.1 mM for growth in the absence of CO_2_/${\text{HCO}}_{3}^{-}$ and transferred to a CO_2_-free atmosphere for 24 h in the presence or absence of inhibitors [NHE-1 inhibitor HOE#694 (Hoechst) 100 μM]. Dishes were then irradiated (0, 2, 4, 6, 8, and 10 Gy). After irradiation, dishes were returned to 5% CO_2_ in regular NaHCO_3_-containing medium for 5 days. Cells were then trypsinized and the percentage of cell death was determined with trypan blue.

### CLONING EFFICIENCY

LS174Tr cells were plated in clonogenic conditions (1000 cells per plate, triplicate) in 25 cm^2^ ventilated flasks during 24 h, then exposed to hypoxia (1% O_2_) for 48 h, and subsequently closed with non-ventilated flask caps before irradiation (0, 1, 2, 4, 6, and 8 Gy). Cells were then returned to normoxic conditions to allow cell recovery and determination of colony number following irradiation. PS120 cells were plated onto 60 mm dishes in clonogenic conditions (1000 cells per dish, duplicate). Once attached, cells were exposed to 30 mM HEPES, 100 μM hypoxanthine, 100 μM uridine, and 10% dialyzed FCS medium adjusted to two different extracellular pH (either pH_o_ 7.0 or 7.5) in a CO_2_/${\text{HCO}}_{3}^{-}$ free environment and subsequently irradiated (0, 1, 2, 4, 6, and 8 Gy) in these environments. Following irradiation, the medium were replaced by a regular medium. Six days (for PS120 cells) or 10 days (for LS174Tr cells) following irradiation, cells were fixed, stained with Giemsa, and counted using ImageJ^®^ software. Of note for both LS174 and PS120 cells, irradiation was not performed but after two or three cell divisions. This raises the possibility of microcolony formation and over-estimation of the number of colonies, however, we used caution to exclude microcolonies from our final analysis. Furthermore, our calculations involve a ratio between experimental clones and control. Therefore, the absolute number of clones is normalized because every condition grows with the same amplitude of error.

### CELL PROLIFERATION IN THREE-DIMENSIONS AND IRRADIATION OF SPHEROIDS

To grow spheroids, 2 × 10^3^ cells were seeded in drops in 20 μl of ${\text{HCO}}_{3}^{-}$-free DMEM buffered with 30 mM HEPES pH 7.7 containing 10% FCS. After 8 days, spheroids were irradiated (0, 2, 4, 6, and 8 Gy) as described above. Intact spheroids were then transferred to polyhema-coated 96-well plates for continued growth in the same respective media for 4 days (12 days total growth time including irradiation). Spheroids were dissociated in Accutase (Life Technologies) and living and dead cells were immediately counted using trypan blue exclusion.

### NUDE MICE XENOGRAFTS AND IRRADIATION OF MOUSE TUMORS

Cells (1 × 10^6^) were subcutaneously injected into the flanks of 4-week-old male athymic *nude* mice (Harlan) according to CNRS institutional guidelines and tumor growth was measured as reported previously (Chiche et al., 2010a). A total of 750 μg/ml doxycycline (DOX; Sigma) was added in the drinking water before the injection of tumor cells following the previously established protocol in our laboratory allowing us to obtain 90% of *ca9* silencing *in vivo* (see Chiche et al., 2010a) for immunohistochemical analysis of inducible *ca9* silencing in this model). Tumors of 4–5 mm were irradiated (8 Gy) with contact X-rays (Gérard et al., 2011) using a RT 50 Phillips unit delivering a 50-kV maximal energy X-ray beam. The source–surface distance was 40 mm and the dose rate was 20 Gy/min. The X-ray tube was handheld and the precision was controlled through direct vision by the radiation oncologist using a 20-mm diameter applicator. The dose was prescribed at the exit surface of the applicator. One single fraction of 8 Gy was delivered into the visible lesion.

### STATISTICAL ANALYSIS

The Student’s *t*-test was used wherein *P*-values of <0.05 were considered significant.

## RESULTS

### INHIBITION OF THE MAJOR pH_i_-REGULATING SYSTEM NHE-1 SENSITIZES CELLS TO RADIATION-INDUCED CELL DEATH

**FIGURE 1:**
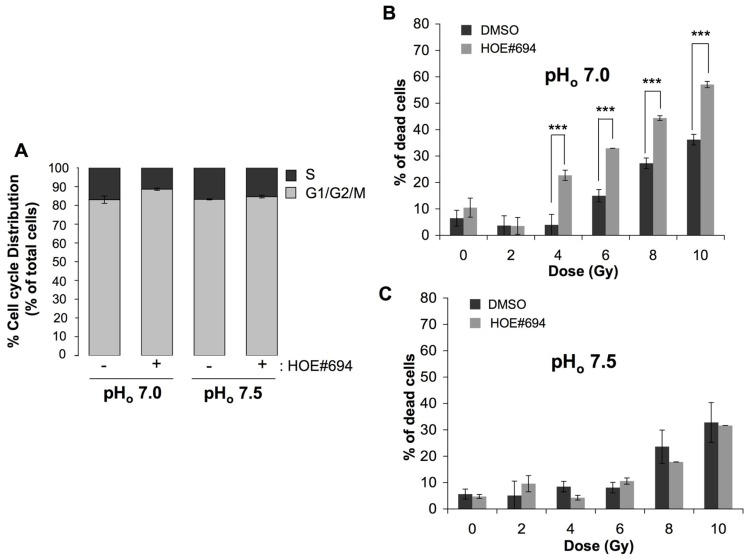
**NHE-1 protects cells against irradiation-induced cell death in an acidic environment.** **(A)** Cell cycle distribution of CCL39 cells treated (+) or not (-) with 100 μM of the NHE-1 inhibitor (HOE#694) in normoxia in a ${\text{HCO}}_{3}^{-}$/CO_2_-free environment at pH_o_ 7.5 or 7.0 for 24 h. **(B,C)** CCL39 cells (1 × 10^4^) were plated in 60 mm dishes. Once attached cells were incubated in a ${\text{HCO}}_{3}^{-}$-free medium adjusted to pH_o_ 7.5 or 7.0, and treated in the presence (+) or absence (-) of 100 μM of NHE-1 inhibitor (HOE#694) for 24 h in a CO_2_-free atmosphere. Dishes were then irradiated (0, 2, 4, 6, 8, and 10 Gy) and returned to a 5% CO_2_ incubator with fresh ${\text{HCO}}_{3}^{-}$-containing medium for 4 days. Cell death was determined by the trypan blue exclusion assay.

The contribution of intracellular acidosis to cell radiosensitivity was studied on fibroblasts growing in pH_o_ 7.5 or a more acidic pH_o_ 7.0 medium, in the presence or in the absence of NHE-1 inhibitor. We choose to work at pH_o_ of 7.0 as it is low enough to reduce the pH_i_ compared to the pH_i_ obtained at pH_o_ of 7.5 but is not low enough to prevent an observation of radiosensitization in acidic conditions due to a reduction in cell viability caused by acidosis alone. The impact of inhibiting NHE-1 on pH_i_ regulation in these cells has been well established with NHE-1 inhibition causing a significant reduction in pH_i_ in a pH_o_ of 7.0 (Pouysségur et al., 1984). Prior to irradiation, we determined the effect of NHE-1 inhibition on cell cycle phase distribution. Selective inhibition of NHE-1 using HOE#694 (100 μM; see Masereel et al., 2003 for a review of NHE inhibitors and HOE#694 effectiveness) at the more acidic pH_o_ 7.0 condition reduced the percentage of CCL39 cells in the most radioresistant S phase (34% decrease of cells in the radioresistant S phase in the presence of HOE#694 compared to non-treated cells) while it had no effect in a more neutral pH_o_ 7.5 medium (**Figure 1A**). Consistent with this finding, irradiation of NHE-1-inhibited fibroblasts in a pH_o_ 7.0 medium led to an increase in cell death (57% for 10 Gy) compared to either untreated cells (35% for 10 Gy; **Figure 1B**) or cells exposed to a pH_o_ 7.5 medium treated or not with HOE#694 (32% for 10 Gy; **Figure 1C**).

### EXPRESSION OF THE HYPOXIA-INDUCED pH_i_-REGULATING CARBONIC ANHYDRASE IX PROTECTS CELLS AGAINST IRRADIATION

**FIGURE 2:**
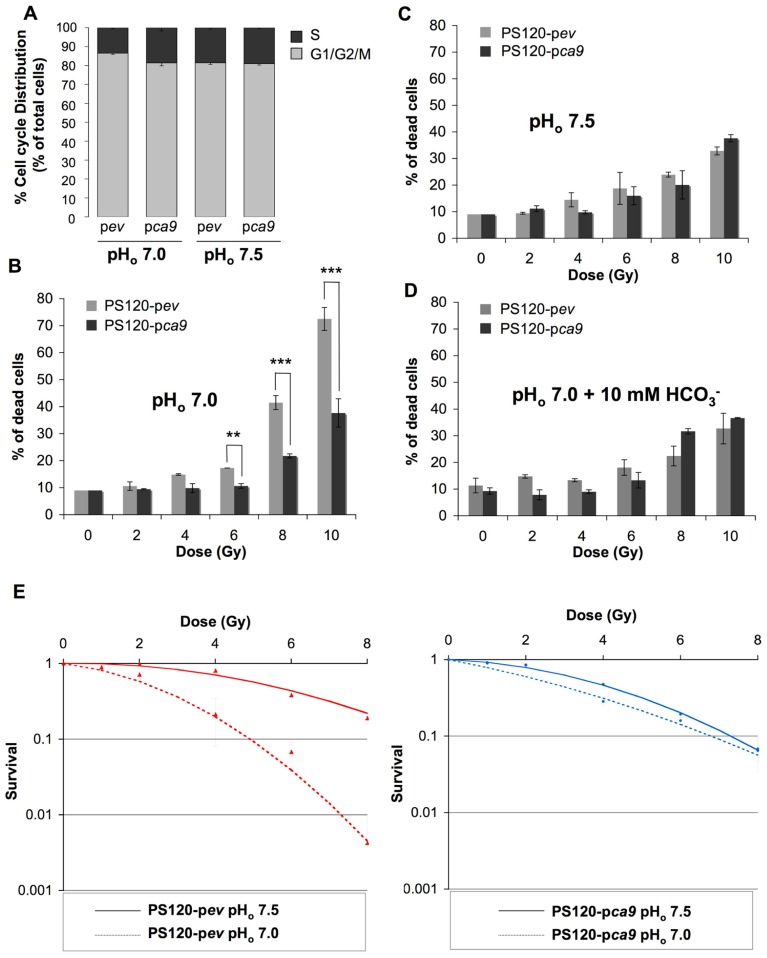
**CAIX protects cells against irradiation-induced cell death in an acidic environment.** **(A)** Cell cycle distribution of NHE-1-disrupted fibroblasts PS120 cells expressing (p*ca9*) or not (p*ev*) CAIX, in normoxia in a ${\text{HCO}}_{3}^{-}$/CO_2_-free environment at pH_o_ 7.0 or 7.5 for 24 h. **(B–D)** PS120-p*ev* and PS120-p*ca9* cells (1 × 10^4^) were plated in 60 mm dishes. Once attached cells were incubated in 30 mM HEPES-buffered ${\text{HCO}}_{3}^{-}$-free medium adjusted to pH_o_ 7.0 in the absence **(B)** or in the presence of 10 mM ${\text{HCO}}_{3}^{-}$ **(D)** or to pH_o_ 7.5 **(C)** for 24 h in a CO_2_-free atmosphere. Dishes were then irradiated (0, 2, 4, 6, 8, and 10 Gy) and returned to a CO_2_-containing incubator with fresh regular ${\text{HCO}}_{3}^{-}$-containing (44 mM) medium for 4 days. Cell death was determined by the trypan blue exclusion assay. Data represent the average of three independent experiments. **(E)** The clonogenic capacity of PS120-p*ev* and PS120-p*ca9* cells exposed to a medium adjusted to pH_o_ 7.0 or 7.5 was measured 10 days after irradiation (0,1, 2, 4, 6, and 8 Gy). Dishes were stained with Giemsa (Fluka). The colonies were counted with ImageJ software according to the following parameters: particles size = 0.15–5 mm^2^ and circularity = 0.1–1.

We have previously demonstrated that expression of catalytically active CAIX in NHE-1-deficient CCL39 fibroblasts (PS120 cells) maintains a higher pH_i_ compared to control PS120 cells lacking CAIX, when cells were exposed to a nominally bicarbonate free acidic medium (Chiche et al., 2010a). Here we showed that in the condition where CAIX is required for pH_i_ regulation (pH_o_ 7.0 compared to pH_o_ 7.5 medium), expression of CAIX in PS120 cells (PS120-p*ca9*) maintains the distribution of the cell cycle phases, while in the absence of CAIX, PS120-p*ev* cells demonstrate a 35% reduction in the most radioresistant S phase (**Figure 2A**). Consequently, control PS120-p*ev* cells growing at pH_o_ 7.0 were shown to be more radiosensitive than PS120-p*ca9* cells, with 70% cell death following irradiation of 10 Gy for PS120-p*ev* cells compared to 37% for PS120-p*ca9* cells (**Figure 2B**). Of note, PS120-p*ca9* cells irradiated with 10 Gy at pH_o_ 7.0 exhibited similar cell death rates to that at pH_o_ 7.5 while PS120-pev cells had much higher cell death at low pH_o_ (**Figures 2B,C**). Thus, active CAIX protects cells against ionizing irradiation at low pH. To definitively validate that the pH_i_-regulating functions of CAIX are indeed involved in cellular radioprotection, PS120-p*ev* and PS120-p*ca9* cells were exposed to a pH_o_ 7.0 medium containing 10 mM ${\text{HCO}}_{3}^{-}$. This ${\text{HCO}}_{3}^{-}$ addition has been shown previously to maintain pH_i_ in acidic pH_o_ environments (Chiche et al., 2010a). Irradiation of PS120-p*ev* cells in the presence of ${\text{HCO}}_{3}^{-}$ reduced the percentage of cell death to that obtained for PS120-p*ca9* cells in a pH_o_ 7.0 medium (**Figure 2D**). Cloning efficiency experiments also confirm the capacity of irradiated cells to survive and recover following irradiation. From 4 to 8 Gy single doses of ionizing radiation of PS120-p*ev* cells exposed to a pH_o_ 7.0 medium drastically reduced the cloning efficiency, compared to that observed in a pH_o_ 7.5 medium (**Figure 2E**, left panel). In contrast, PS120-p*ca9* cells exposed to a pH_o_ 7.0 medium were capable to recover after irradiation, to the same extent that we observed in a pH_o_ 7.5 (**Figure 2E**, right panel). Taken together these results suggest that the pH_i_-regulating properties of NHE-1 and CAIX protect cells against irradiation.

### DUAL SILENCING OF THE HYPOXIA-INDUCED pH_i_-REGULATING-SYSTEM *ca9/ca12* STRONGLY COMPROMISES *IN VITRO* AND *IN VIVO* TUMOR GROWTH WHEN COMBINED WITH IONIZING RADIATION

**FIGURE 3:**
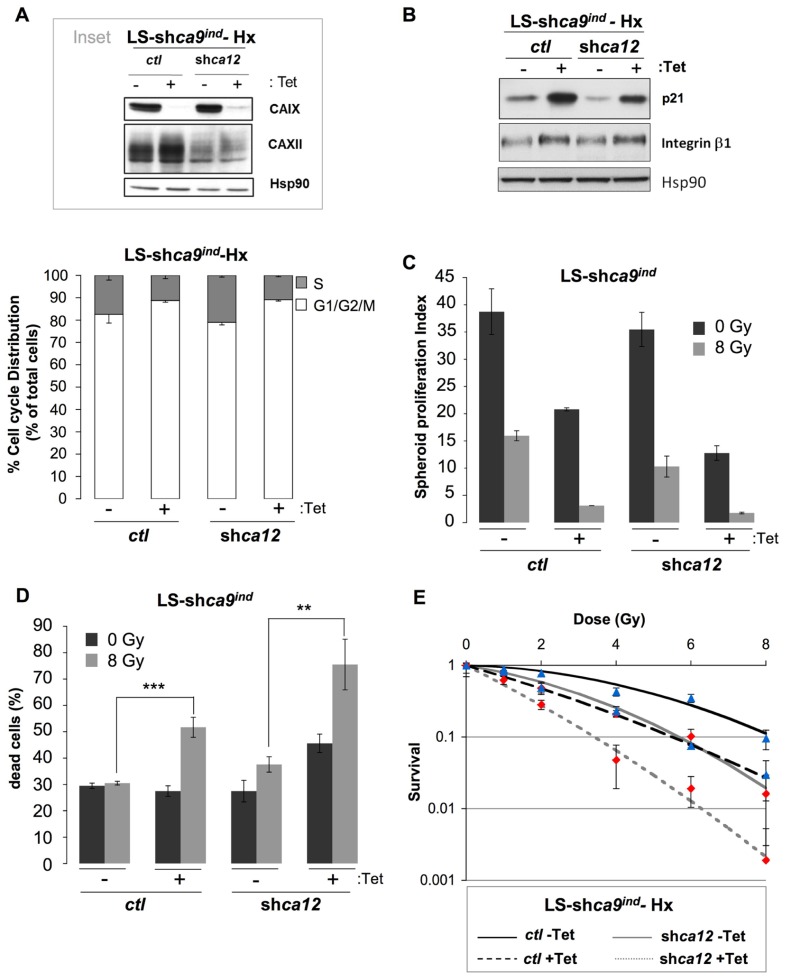
**Silencing of the hypoxia-induced pH_i_-regulating enzymes *ca9* and *ca12* induced *in vitro* cell death of LS174Tr cells when combined with ionizing radiation.** **(A)** *Inset*: Expression of CAIX and CAXII in colon carcinoma Tetracycline (Tet)-inducible LS-sh*ca9/ctl *-Tet cells either silenced for *ca9* (LS-sh*ca9/ctl* +Tet) or for *ca12* (LS-sh*ca9/ca12*^-^ -Tet) or both *ca9* and *ca12* (LS-sh*ca9/ca12*^-^ +Tet) in hypoxia 1% O_2_ (Hx) for 48 h. Hsp90 was used as a loading control. The cell cycle phase distribution was determined by FACScan analysis of LS-sh*ca9/ctl *-/+Tet and LS-sh*ca9/ca12*^-^ -/+Tet cells exposed hypoxia of 1% O_2_ (Hx) for 24 h in a ${\text{HCO}}_{3}^{-}$-containing medium. **(B)** Immunoblotting of p21, β1 integrin, and Hsp90 (loading control) in LS-sh*ca9/ctl* and LS-sh*ca9/ca12*^-^ cells pre-incubated for 4 days in the presence (+Tet) or absence (-Tet) of Tet to silence *ca9*, before exposure to hypoxia of 1% O_2_ (Hx) for 48 h (H). **(C,D)**. Tet-inducible LS174Tr cells silenced for *ca9* or *ca12* or both *ca9* and *ca12* were cultured as spheroids in a CO_2_ atmosphere and HEPES-buffered ${\text{HCO}}_{3}^{-}$-free medium (pH_o_ 7.7 in the absence (-Tet) or presence (+Tet) of Tet for 8 days before they were irradiated (8 Gy) or not (0 Gy). After irradiation, spheroids were transferred to polyhema-coated 96-well plates containing fresh medium for 5 days. Spheroids were then subjected to Accutase dissociation and individualized live cells **(C)** and dead cells **(D)** were counted using trypan blue. The spheroid proliferation index was calculated as the ratio of the number of living cells counted at day 13 to the number of cells at day 0. Data represent the average of three independent experiments. **(E)** The clonogenic capacity of LS174Tr-sh*ca9/ctl* -/+Tet and LS174TR-sh*ca9/ca12*^-^ -/+Tet cells exposed to hypoxia (1% O_2_) for 48 h in a regular medium, was measured 10 days after irradiation (0,1, 2, 4, 6, and 8 Gy). Dishes were stained with Giemsa (Fluka). The colonies were counted with Image J software.

LS174Tr cells cultured in hypoxia before exposure to an increasing dose of ionizing radiation demonstrated a higher cloning efficiency than normoxic cells along with equal distribution of cell cycle phases before irradiation (data not shown). This established the classical radioresistance of LS174Tr cells as observed in other hypoxic cells and validated this model for our study. In a regular ${\text{HCO}}_{3}^{-}$-containing medium a higher number of cells in the radiosensitive G1/G2/M phases was observed when *ca9* or both *ca9* and *ca12* were silenced (**Figure 3A**). Protein expression levels of CAIX and CAXII in the Tet-inducible silencing of *ca9* in control LS174Tr cells (LS-sh*ca9*/*ctl*) or *ca12* silenced cells (LS-sh*ca9*/*ca12*^-^) were confirmed for efficient knock-down (**Figure 3A**, inset). In the same conditions, *ca9* or both *ca9*/*ca12* silencing was accompanied by an increase in p21, E-cadherin, and β1 integrin expression, which were associated with a reduced cell proliferation (**Figure 3B**). To mimic both the tumor hypoxic and proton gradient observed *in vivo*, we grew LS174 cells in three dimensions. Spheroids were grown in nominally bicarbonate free media to enhance the pH gradients that develop during spheroid growth. Irradiation of *ca9*-silenced spheroids (LS-sh*ca9*/*ctl* +Tet, 8 Gy) revealed a cumulative decrease in the proliferation index (**Figure 3C**) and a twofold increase in cell death from 27.5% (0 Gy) to 51.7% (8 Gy) when compared to non-irradiated *ca9*-silenced spheroids (**Figure 3D**). While *ca12* silencing alone did not alter the proliferation rate of non-irradiated spheroids, irradiation of *ca12*-silenced cells (LS-sh*ca9*/*ca12*^-^ -Tet 8 Gy) reduced the proliferation index (**Figure 3C**) and increased cell death from 27.5% (0 Gy) to 37.6% (8 Gy; **Figure 3D**). Irradiation of double silenced cells (LS-sh*ca9*/*ca12*^-^ +Tet 8 Gy) strongly compromised proliferation and viability (75.5% cell death; **Figures 3C,D**). Clonogenic test confirmed that double silenced cells (LS-sh*ca9*/*ca12*^-^ +Tet) exposed to hypoxia were less capable to recover from irradiation compared to control cells (LS-sh*ca9*/*ctl* -Tet) or single *ca9* or *ca12*-silenced cells (LS-sh*ca9*/*ctl* +Tet and LS-sh*ca9*/*ca12*^-^ -Tet; **Figure 3E**).

**FIGURE 4:**
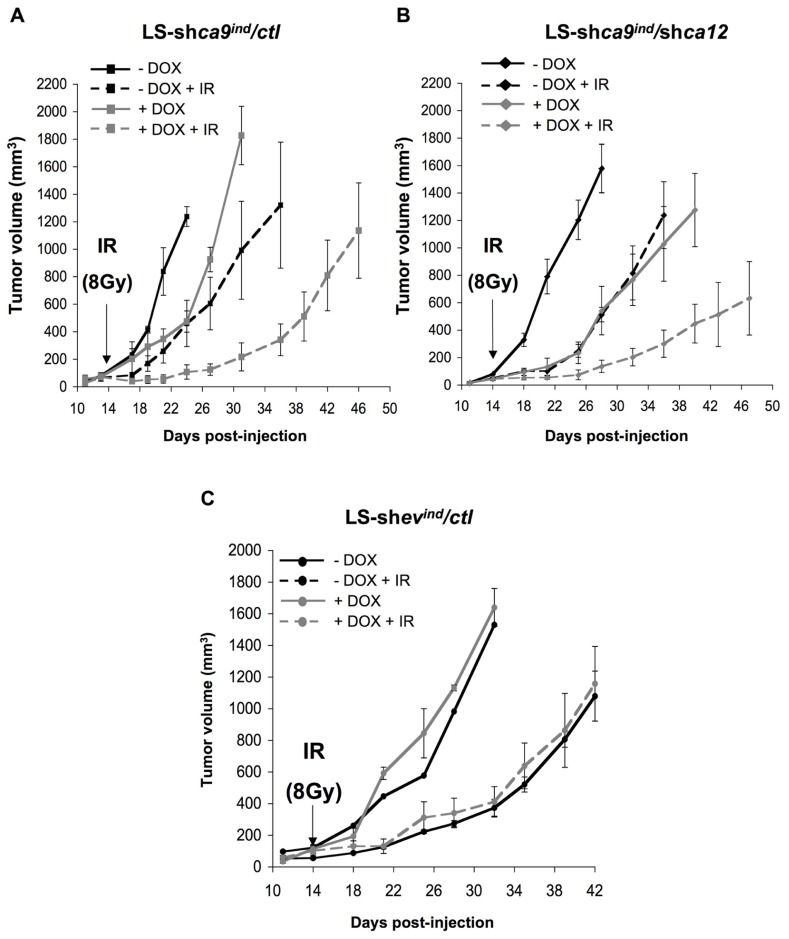
**Combined silencing of *ca9* and *ca12* compromises *in vivo* LS174Tr cell proliferation when combined with ionizing radiation.** **(A,B)**. Anti-tumor activity of silencing *ca9* or *ca12* individually or combined is increased in conjunction with ionizing radiation in LS174Tr xenograft tumors. At 4 days before injection of LS-sh*ca9*/*ctl* **(A)** or LS-sh*ca9/ca12*^-^ cells **(B)**, cells were incubated with or without Tet (+/-Tet) to silence *ca9*. *In vivo* xenograft assays were performed by s.c injection of viable and individual tumor cells (1 × 10^6^) into the flanks of athymic *nude* mice. To maintain *ca9* knock-down mice received DOX in the drinking water 4 days before cell injection. Fifteen days after cell injection, when the tumor size reached 4–5 mm, a single dose (8 Gy) of irradiation (IR) was delivered to the tumors only with “contact X-rays.” Xenograft growth was determined by measuring the tumor volume. **(C)** Xenograft tumor growth of control tumors LS-sh*ev/ctl* irradiated (IR) or not, in the presence (+DOX) or in the absence (-DOX) of doxycycline in the drinking water. Five mice were used per condition.

Using contact radiotherapy (Gérard et al., 2011), we specifically targeted the established tumor mass grown on the back of *nude* mice. Irradiation of control tumors (LS-sh*ca9*/*ctl* -DOX + IR) stopped tumor progression for 5 days after irradiation before proliferating again at a high rate, which was similar to that of non-irradiated control tumors (LS-sh*ca9*/*ctl* -DOX; **Figure 4A**). Tumor progression was delayed with *ca-9*-silencing as observed previously while irradiation of *ca9*-silenced tumors (LS-sh*ca9*/*ctl* +DOX +IR) showed more pronounced arrest in tumor progression (25 days after irradiation to reach 600 mm^3^), which may reflect cell death within the tumor (**Figure 4A**). Irradiation of *ca12*-silenced tumors (LS-sh*ca9*/*ca12*^-^ -DOX +IR) reduced the growth rate compared to non-irradiated tumors (LS-sh*ca9*/*ca12*^-^ -DOX) to the same extent observed for irradiation of control tumors suggesting that *ca12* alone is not able to confer tumor radioresistance (**Figure 4B**). However, irradiation of double silenced tumors (LS-sh*ca9*/*ca12*^-^ +DOX +IR) showed a substantial reduction in the progression of the tumor (33 days after irradiation to reach 600 mm^3^). No interaction between DOX and irradiation was observed as shown with control LS-sh*ev*/*ctl* -/+DOX tumors (**Figure 4C**). Calculations of the tumor growth delay for time required to reach 300 and 600 cm^3^, respectively were 17.6/19.7 days (LS174shCA9 -Dox, -IR), 25.7 days/29.1 days (LS174shCA9 -Dox, +IR), 25.7 days/29 days (LS174shCA9 +Dox, -IR) and 36 days/46.3 days (LS174shCA9 +Dox, +IR). Growth delay times for CA9/CA12 double-silencing were 33.9 days/39.9 days (LS174shCA9/CA12 +Dox, -IR) and 36.1 days/45.9 days (LS174shCA9/CA12 +Dox, + IR). We further calculated the specific tumor growth delay (STGD) with the following formula: STGD = (DT experimental - DT control)/DT control (DT, doubling time). Irradiation alone and CA9 silencing alone resulted in similar STGD values of 0.61 and 0.57, respectively. Combined irradiation and silencing of CA9 increased the STGD to 3.9 compared to control. Meanwhile silencing of CA12 resulted in a STGD value of 1.85 while double CA9 and CA12 silencing with irradiation had a similar STGD value to CA9 silencing of 3.67.

These results demonstrate that silencing of *ca9* and double-silencing of *ca9*/*ca12* combined with ionizing radiation substantially decreases tumor growth in both *in vitro* and *in vivo* model systems.

## DISCUSSION

Acidosis within the tumor microenvironment arises from the hypoxia-induced metabolic shift from oxidative phosphorylation to glycolysis, along with the capacity of hypoxic tumor cells to regulate their pH_i_ through efficient export of CO_2_ and lactic acid. Consequently, targeting tumor pH_i_-regulating systems in hypoxia holds potential as a key strategy to reduce tumor growth (Pouysségur et al., 2006). Here we have explored a combination of this pH disrupting strategy with radiotherapy as it was suggested that acidosis plays a role in tumor radioresistance (Vaupel, 2004). However, previously there was no clear demonstration whether the extracellular and/or the intracellular acidosis were responsible for the poor radioresponse of tumor cells. Dubois et al. (2011) have demonstrated that a combination of CAs inhibition with irradiation in colon HT29 tumor-bearing mice improved the anti-tumor effect compared to a single radiation dose. However, CA inhibition did not result in increased radiosensitivity *in vitro* and the contribution of the pH-regulating functions of CAIX in the tumor radio-response remained to be clarified. Herein, we demonstrate the importance of pH_i_ regulation in radioresistance by observing an increase in radiation-induced cell death of fibroblasts inhibited for NHE-1 or lacking both NHE-1 and CAIX when they are grown in an acidic and ${\text{HCO}}_{3}^{-}$-free medium. In contrast, ectopic expression of CAIX was able to improve cell survival following irradiation. The mechanism of CAIX-induced radioresistance was demonstrated with NHE-1-deficient CCL39 fibroblasts (PS120 cells) in a nominally CO_2_/${\text{HCO}}_{3}^{-}$ free acidic environment by: (i) a decrease in the pH_i_-regulating capacity of cells lacking CAIX (see Chiche et al., 2009) and (ii) a correlation with the positioning of these cells in the most radiosensitive G1/G2/M phases, prior to irradiation. This cell cycle data is consistent with the reduction in S phase entry as previously demonstrated for PS120 cells compared to the parental cell line (Pouysségur et al., 1985). Expression of CAIX prevents the reduction of cells in S phase as it allows cells to maintain a higher pH_i_ in acidic medium (Chiche et al., 2010a). With addition of ${\text{HCO}}_{3}^{-}$/CO_2_ in low pH medium (pH_o_ 7.0) we observed no difference in cell death between irradiated-PS120-p*ev* and irradiated-PS120-p*ca9* cells due to the buffering ability of ${\text{HCO}}_{3}^{-}$/CO_2_ to restore alkaline pH_i_ values as previously demonstrated (Chiche et al., 2010a). We conclude that the CAIX-induced protection against irradiation at pH_o_ 7.0 could be explained by the capacity of CAIX to sustain an intracellular alkaline shift.

Under three-dimensional growth conditions that result in hypoxia (Chiche et al., 2010a; Pelletier et al., 2012) and acidosis (Swietach et al., 2010), LS174Tr spheroids silenced for *ca9*/*ca12* showed a decrease in proliferation and a cumulative increase in cell death (75%) after a single radiation dose. Double silenced cells were indeed most sensitive to irradiation, since: (i) combined silencing reduced the capacity of LS174Tr cells to regulate their pH_i_ in acidic medium while single silencing of *ca9* was not sufficient to do so (Chiche et al., 2010a), (ii) silencing of *ca9*/c*a12* increased p21 expression indicating a cell cycle arrest in G1 along with increased levels of β1 integrin, two key proteins involved in cell contact and adhesion which may influence proliferation (Svastová et al., 2003) and (iii) *ca9* silencing lead to a reduction in proliferation and a decrease in cell number in the radioresistant S phase. A single radiation dose on xenograft tumors dramatically reduced the growth rate of *ca9*- and *ca9*/*ca12*-silenced tumors. Twenty-five days after irradiation, *ca9*-silenced tumors recovered a growth rate that was comparable to control tumors, while *ca9*/*ca12*-silenced and irradiated tumors never recovered the growth rate of control cells. In addition, the loss of radioresistant hypoxic cells due to CA9/CA12 silencing could contribute to the reduction of tumor growth in combination with their radio-sensitivity due to decreased pH_i_ regulation.

Hypoxia-specific cytotoxins such as tirapazamine form toxic radical species that act to kill hypoxic cells and are thus proposed to be used in combination with irradiation to create a synergistic effect (Brown, 1993). Unfortunately this treatment-strategy failed to be efficient in patients (Rischin et al., 2010). Intense research is ongoing in the development of small molecule inhibitors to specifically target membrane-bound CA(s) over cytosolic CAs to appraise the potential of targeting CAIX and CAXII to decrease tumor progression (Morris et al., 2011). The synthesis of new CA(s) inhibitors (Supuran, 2008; Morris et al., 2011) has also revived interest in acetazolamide (ACTZ), which has been used in the clinic for over 40 years as a CA inhibitor (Kaur et al., 2002). Recently, ACTZ was linked to a C-terminal albumin-binding peptide (Albu-ACTZ) with the aim of not only reducing blood clearance but also preventing internalization of the molecule to target more specifically membrane-bound CAIX and CAXII. This compound demonstrated its *in vivo* efficacy by retarding tumor growth of renal SK-RC-52 xenografts. However, it had no significant impact on highly proliferative LS174Tr tumors (Ahlskog et al., 2009).

In the present study, we took advantage of the expression of the hypoxia-induced pH_i_-regulating systems CAIX and CAXII to target radioresistant hypoxic cells. This study reinforces the notion that CAIX and CAXII represent potential targets for anti-cancer treatment. The present study also supports the use of radiotherapy in combination with CAs inhibition as a new anti-cancer strategy.

## Conflict of Interest Statement

The authors declare that the research was conducted in the absence of any commercial or financial relationships that could be construed as a potential conflict of interest.

## ACKNOWLEDGMENTS

The laboratory is funded by the Ligue Nationale Contre le Cancer (Equipe labellisée), the EU-FP7-“METOXIA,” the Association pour la Recherche contre le Cancer, the Institut National du Cancer, the Agence Nationale pour la Recherche, the Centre Antoine Lacassagne, the Centre National de la Recherche Scientifique, the Institut National de la Santé et de la Recherche Médicale, and the University of Nice. Scott K. Parks was funded by The Natural Sciences and Engineering Research Council of Canada (NSERC) and the Association pour la Recherche contre le Cancer (ARC). Johanna Chiche was funded by the “METOXIA” grant and the ARC.
